# Ischemic Brain Stroke and Mesenchymal Stem Cells: An Overview of Molecular Mechanisms and Therapeutic Potential

**DOI:** 10.1155/2022/5930244

**Published:** 2022-05-25

**Authors:** Yang Jingli, Wang Jing, Yasmeen Saeed

**Affiliations:** ^1^Provincial Key Laboratory for Utilization and Conservation of Food and Medicinal Resources in Northern Guangdong, 288 University Ave. Zhejiang District, Shaoguan, Guangdong Province, China; ^2^Department of Neurology, The First Affiliated Hospital of Harbin Medical University, 157 Baojian Road Harbin, Heilongjiang, China 150081; ^3^Guangdong VitaLife Biotechnology Co., Ltd. No. 61 Xiannan Road, Nanhai District, Foshan, Guangdong, China 528200

## Abstract

Ischemic brain injury is associated with a high rate of mortality and disability with no effective therapeutic strategy. Recently, a growing number of studies are focusing on mesenchymal stem cell-based therapies for neurodegenerative disorders. However, despite having the promising outcome of preclinical studies, the clinical application of stem cell therapy remained elusive due to little or no progress in clinical trials. The objective of this study was to provide a generalized critique for the role of mesenchymal stem cell therapy in ischemic stroke injury, its underlying mechanisms, and constraints on its preclinical and clinical applications. Thus, we attempted to present an overview of previously published reports to evaluate the progress and provide molecular basis of mesenchymal stem cells (MSCs) therapy and its application in preclinical and clinical settings, which could aid in designing an effective regenerative therapeutic strategy in the future.

## 1. Introduction

Ischemic stroke is attributed to a high disability rate with limited or no therapeutic options for functional recovery [1–3]. Intrinsically, ischemic stroke indicates the cascade of congesting events, i.e., thrombus formation and embolism, that ultimately decreases the local blood flow and cause oxygen deprivation in affected brain tissue. Besides, systemic hypoperfusion is another major factor in the occurrence of ischemic stroke [4].

According to the World Health Organization (WHO), ischemic stroke is the most ubiquitous subclass of stroke that affects almost 15 million people globally [5]. However, to date, available treatment preferences are largely precautionary in purview [6]. For instance, thrombolytic agents such as tissue plasminogen activator (tPA) exhibit limited or no physical recovery of patients suffering from stroke [6, 7]. Surgical interference can only aid in minimizing the risk of clot formation [8]. Therefore, an effective therapeutic strategy is required to prevent the onset of acute stroke and manage the chronic symptoms associated with neural ischemia, i.e., long-term neuroinflammation and localized necrosis [8, 9].

The recent decade has seen encouraging outcomes of mesenchymal stem cell therapy that holds promise to alleviate the burden of neurological disorders Moreover, initial study data of preclinical trials have also indicated the effectiveness, tolerance, and safety of MSC-based therapy [10]. Thus, MSCs were suggested as a promising candidate for ischemic brain injury patients[11]. However, effective dose and appropriate time of MSCs delivery are the main challenges in the clinical translation of stem cell therapy. Therefore, a carefully designed, future study plan is a prerequisite for randomized clinical application trials to estimate its functional clinical outcome [10].

Herein, we presented an overview of a previously published work regarding the role of stem cell therapy in ischemic stroke and its underlying molecular mechanisms. We aim to provide the basis for establishing a future study to promote the clinical translation of stem cell therapy in ischemic brain diseases.

## 2. Significance of Therapeutic Application of MSCs in Ischemic Stroke

**Figure 1:**
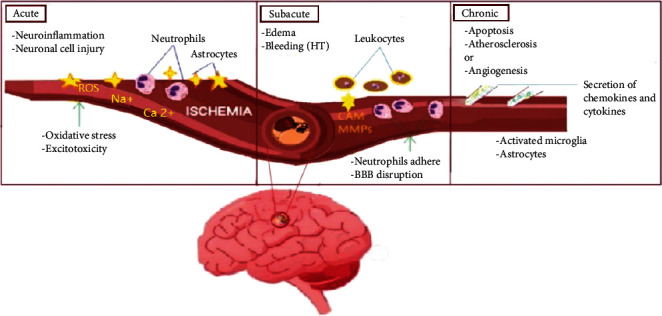
Graphical presentation of the various stages of cerebral ischemia stroke, i.e., acute phase, subacute phase, and chronic phase and their contributing factors.

An insight into the biology of ischemic stroke indicates that a stream of molecular events initiates instantly after the onset of ischemic stroke, such as oxidative stress, increased level of intracellular calcium, excitotoxicity, and inflammation which results in apoptotic or necrotic neuronal cell death [12–14]. According to previously established studies [8], the ischemic avalanche followed by a stroke is comprised of three phases, i.e., (i) acute phase, (ii) the subacute phase, and (iii) the chronic phase (Figure 1). Further insights into its molecular events indicate that the acute phase takes almost 2 weeks to complete after the incidence of the injury [15]. The subacute or secondary phase continues its deleterious events up to 6 months after the onset of the lesion. The duration of the chronic phase could take months to years after stroke and may last for the rest of the patient's life along with its neuro-damaging sequel [15].

Briefly, the biology of stroke indicates the role of inflammation in the parthenogenesis of stroke, which collectively points towards disruption of ionic balance, oxidative stress, and deregulation of signaling pathways that ultimately overburden astrocytes and results in transient hyperglycolysis and calcium influx while accumulating a high concentration of lactate in the extracellular fluid [16]. For instance, a recent study by Sarah Martha's article shows that molecules that control acid-base balance and electrolytes possess the potential to be effective therapeutic targets to preserve neurons in the ischemic brain [17].

Glial scar formation is another major hurdle in axon regeneration which ultimately exaggerates the inflammatory response and chronic pain [16]. Intriguingly, natural killer (NK) cells can develop infarction by secreting IFN-*γ* in T- and B-cell-independent mechanisms and stimulate local inflammation by secreting pro-inflammatory cytokines such as IFN-*γ*, IL-17a, TNF-*α*, IL-1*β*, IL-6, IL-12, and ROS after middle cerebral artery occlusion (MCAO) [16]. Besides, further studies have indicated the presence of activated T-cells, 60 days after the onset of the injury. Among T-cells, increased expression of CD4+ CD25+ FoxP3+ Treg cells and CD4+ CD28*−* T-cells are noticed in stroke patients [16].

It is also important to note that the recently available therapeutic strategy for acute ischemic stroke depends on reperfusion via endovascular or thrombolytic therapy [18]. However, limited therapeutic aperture for the administration of the thrombolytic agent tPA (<4.5 hours from symptom onset) and aggravation of blood-brain barrier (BBB) break down are drawbacks of these strategies [19–21]. Therefore, despite an increasing number of studies concerning the etiology, pathophysiology, and treatment of ischemic stroke [22–24], the range of therapeutic interventions has remained very limited [14, 25].

Nonetheless, stem cell therapeutic strategies have shown the potential to combat the deleterious effects of acute, subacute, and chronic phases of ischemic stroke [6]. Moreover, preclinical studies have demonstrated the safety of stem cell therapy against ischemic stroke by evaluating the possible therapeutic outcome [11]. Further studies have indicated that MSCs derived from umbilical cord lining (UC-MSCs) are profoundly immunological immature cells, and this property makes them a promising candidate for the treatment of stroke [26, 27]. For instance, UC-MSCs can potentially reduce the infarct size and ameliorate the functional recovery by elevating the expression of growth and neuroprotective factors such as brain-derived neurotrophic factor (BDNF) and vascular and endothelial growth factor (VEGF) [28–33]. Given these above-mentioned properties, MSCs have been designated as “Advanced Therapy Medicinal Products (ATMP)” according to the guidelines from the American Code of Federal Regulation of the Food and Drug Administration and the European Medicines Agency and the network of national agencies [34].

However, despite being regarded as potential therapeutic candidates for neurological disorders, the clinical application of mesenchymal stem cells has been challenged by poor migration of cells towards the injured site and low survival rate [35, 36]. Another constrain indicates that only a low percentage (<10%) of transplanted MSCs differentiate or express neuronal markers, i.e., NeuN and MAP-2 [32–35]. Given these limitations in therapeutic approaches, we attempted to investigate the role of mesenchymal stem cell therapy in neuroprotection and analyze it in light of previously reported studies.

## 3. Role of Stem Cell Therapy in Ischemic Stroke Recovery and Its Underlying Mechanisms

A growing number of studies have attempted to devise an effective therapeutic modality by considering the pathophysiology and underlying molecular mechanism of stroke [37]. Besides, recent studies have indicated the role of lncRNAs (long noncoding RNAs) in poststroke brain damage, thus suggesting a novel therapeutic target for stroke patients [38]. Stem cells and resident progenitors play an immense role in neuroplasticity after strokes, by the release of the growth factors and exosomes which accelerate post-stroke recovery [39]. Thus, stem cell therapy was suggested as a promising strategy for stroke and other neurological conditions. However, there is further comprehensive understanding of its molecular mechanism to unravel the intrinsic signaling pathways through which stem cells cooperate with the pathophysiology of stroke patients.

The efficiency of stem cell therapy is mainly attributed to the effective crossing of BBB to reach the target site in the brain [2]. Thus, based on previously reported studies, three hypotheses suggest the underlying neuroprotective mechanism of stem cell therapy. The primary hypothesis implies that MSCs inflect the immune system to inhibit the damaging effects of possible autoreactive responses and protect the central nervous system (CNS) [40]. Moreover, thrombosis and hypoxia trigger an intravascular inflammatory cascade, which further augments the innate immune response to cellular damage in the parenchyma and results in secondary tissue injury [41], although the role of adaptive immunity in the pathogenesis of stroke and its long-term effects on the postischemic brain remained elusive. However, a persistent autoimmune response to brain antigens is a major damaging and long-lasting factor. Thus, it was suggested that immunity has long-term outcomes after stroke [41]. The second hypothesis emphasizes the secretion of neuroprotective factors by MSCs, which further trigger the innate repairing mechanisms of the central nervous system and negate the proceeding of neuronal tissue degeneration. For instance, stem cell treatment demonstrates apparent beneficial effects in preclinical stroke models by reducing infarct size and improving behavioral and histological deficits [42] by secreting the growth-promoting factors glial cell line-derived neurotrophic factor (GDNF) and brain-derived neurotrophic factor (BDNF). For instance, accumulating evidence indicates that combined application of cell therapy with growth factors could significantly enhance the stimulation of endogenous neurogenesis, anti-inflammation, neuroprotection properties, and enhancement of stem cell survival rates that potentially contribute to functional recovery [42].

The third hypothesis indicates that transdifferentiation of MSCs into brain cells results in cell replacement; however, most studies do not support this dogma [43], though the transdifferentiation potential of adult stem cells, their capacity for tissue renewal, and damage repair potential have attracted the attention of biotechnologists and clinicians[7]. However, the isolation and maintenance of stem cells are the main challenges for practical application [44]. There is also a possibility that studies providing the dogma of transdifferentiation of MSCs may not necessarily be MSCs, but it could be the progenitor and differentiated cells that escape the immune system surveillance after the onset of injury and survive in the CNS [45]. Hence, the claim of in vitro transdifferentiation of MSCs into neural lineage can not be proved by in vivo evidence [45].

Besides, stem cells have been reported to promote nerve recovery either by the nerve repair strategy (injecting cells to the injury site) or by the immunomodulatory function. Stroke-relevant conditions such as deoxygenation and glucose deprivation can also be applied in combination with cocultured immune cells to study its impacts on its structural, functional, and expressional changes in the system [16]. Moreover, pharmacological targeting of AMP kinase activity, which is known to block microglia/macrophages M1 polarization, appears promising to improve stroke recovery in chronic kidney disease (CKD). Neurogenesis, which allows replacing damaged neurons, also favors stroke recovery [46].

**Figure 2:**
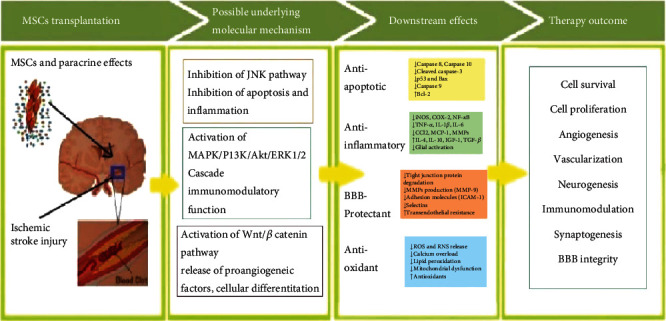
Schematic overview indicating the effects of mesenchymal stem cell (MSC) based cell therapy in ischemic stroke recovery by demonstrating the underlying mechanism and downstream signaling factors.

Besides, immunomodulatory strategies determine the potent role of hypoxia in stem cell therapy via cell migration towards the target site [47], particularly regarding ischemic stroke. Concordantly, hypoxic preconditioning enhances the expression of angiogenic factors, peculiarly, VEFG, which further trigger the functional role of MSCs in repairing the infarcted myocardium [48, 49]. Further studies have suggested the association of the hypoxic microenvironment with cerebral ischemia, which further promotes the migration of UC-MSCs and incites their angiogenic properties via promoting their differentiation into vascular cells and enhancing the release of angiogenic factors. Besides, a low-oxygen environment elevates the expression of migration-related signaling factors and engraftment in UC-MSCs [50]. Accordingly, a preclinical study has suggested hypoxia as a prominent player in stimulating UC-MSCs to minimize neurological defects and promote angiogenesis in the brain of the rat ischemic stroke model. MSC-based therapy could potentially reduce the inflammatory response and neuronal cell apoptosis by modulating the immune system and impeding the secondary damage after ischemic stroke [14, 51, 52]. Accordingly, Figure 2 indicates the underlying molecular pathways through which MSCs counter the damaging effects of ischemic stroke in the brain (Figure 2). Another study has stated that the administration of stem cells during the subacute phase avert early cell death by curbing apoptosis, oxidative stress, mitochondrial impairment, and inflammation.

Furthermore, an elevated level of cytokines or growth factors (in case of ischemic brain injury) results in activation of survival signaling mechanisms in neurons such as MAPK/Erk1,2 pathways [53], while MSCs also aid in the upregulation of PI3-kinase resulting in the activation of Akt signaling and phosphorylation in neuronal cells, thus regulating neuronal survival or death [54]. Further investigations indicate that MSC-derived neurotrophic growth factors could be the main reason for stimulating the upregulation of PI3-K/Akt and MAPK survival signaling pathways in neurons. For instance, BDNF promotes the activation of the receptor tyrosine kinases (Trk), which results in the downstream stimulation of PI3-K/Akt and Erk1,2 signaling pathways and ultimately aid in the survival and differentiation of neurons [55].

Further insight into the underlying molecular mechanism has revealed that activation of the c-Jun N-terminal Kinase pathway (JNK) possesses a significant role in neuronal apoptosis during ischemic stroke [56]. Besides, JNK signaling is considered the preeminent factor regulating neuronal inflammation as focal cerebral ischemia and reperfusion (I/R) proceed [57, 58]. Inhibition of JNK activation could suppress glial cell inflammation and/or neuronal apoptosis, which results in neuroprotection [59]. Besides, the release of paracrine factors is another significant advantage of MSCs that not only promote the survival of astrocytes but also inhibit p38 MAPK and JNK by downregulating the expression of Glial Fibrillary Acidic Protein (GFAP) [60]. Accordingly, a study using the ischemic stroke mouse model has shown that intravenous intervention of bone marrow mesenchymal stem cells (BMSCs) inhibits the JNK signaling pathway and results in the reduction of neuronal cell apoptosis and modulation of the inflammatory response in the ischemic brain [14]. Moreover, BMSCs have been demonstrated to release a combination of numerous growth factors and cytokines that further stimulate the innate survival signaling pathways, including MAPK/ERK1/2 and the PI3K/Akt cascade [61]. Nonetheless, the neuroprotective effect of inhibiting the nuclear factor kappa-light-chain enhancer (NF-*κ*B) of activated B cells has been reported to minimize the damaging effects of inflammation at the injured site [2, 62]. Collectively, the above-mentioned studies speculate that targeting inflammatory pathways via hindering the signaling cascade is the most commonly accepted potential strategy used by MSCs to minimize neurological damage (Figure 2).

The effectiveness and efficiency of stem cell therapy directly depend on the efficient crossing of the BBB by stem cells to sustain the probity of the BBB and to reach the targeted injured brain site [2]. Therefore, the regulation of BBB-specific mechanisms is the main factor in the success of MSC therapy. The evolutionary conserved canonical Wnt pathway (referred to as the Wnt/*β*-catenin pathway) [63] orchestrates BBB formation and maturation during ontogeny [21, 64]. Although the canonical Wnt pathway only has a nominal function in matured brain vasculature, it is vital for the maintenance of BBB integrity [65]. Activation of the canonical Wnt pathway establishes a clinically admissible approach to broaden the therapeutic efficiency by constricting the BBB breakdown and regulation of BBB-specific mechanisms [21]. Moreover, the canonical Wnt/*β*-catenin pathway stimulates the differentiation of MSCs into type II alveolar epithelial cells, which further huddles defiance against oxidative stress and boosts the MSC's migration. Collectively, it was indicated that the Wnt/*β*-catenin pathway could be a crucial mechanism to augment the therapeutic effect of MSCs [8]. Yet, the precise mechanism (through which the Wnt/*β*-catenin pathway) enhancing the therapeutic effect of MSCs remains to elucidate[66].

Besides, the transcriptomic analysis has revealed the crucial of target genes, such as BCL2A1 and TPM2, in the pathogenic mechanism of ischemic stroke [64–67]. Nonetheless, future advancement in the creation of reliable human brain model systems in vitro holds the potential to improve high-throughput screening platforms and provide stroke researchers with a mechanism to screen large numbers of potential drug targets [16].

## 4. *In Vivo* Study Models of MSCs for Ischemic Stroke

Experimental data from *in vitro* ischemia models has shown that even short-term exposure of MSCs on neuronal cell survival at the injured site prominently decreases the detrimental effects of the inflammatory cascade [68]. Accordingly, intracerebral transplantation of MSCs in a rat model of stroke has been reported to induce neuronal activity by stimulating angiogenesis, reducing cell death, and immune response modulation [69, 70]. This property could be attributed to the release of paracrine factors by MSCs, peculiarly TGF-*β*, which reduce the CD68+ cell infiltration by inhibiting MCP-1 secretion [71, 72]. Intriguingly, another study reported a significant increase in the number of axons in a rat model of brain ischemic stroke, which continued even after one year of intra-arterial transplantation of MSCs [73]. Moreover, intra-arterially transplanted MSCs in the rat brain injured by stroke have been reported to reduce the IL-6 mRNA and IL-2 mRNA levels, thereby modulating the immune response [74].In addition to mice or rat models for ischemic stroke injury, larger animals, i.e., dogs and swine, have also been tested for MSC transplantation to get more reliable data for clinical application. Accordingly, UC-MSCs have been reported to enhance the expression of biomarkers for astrocytes and neurons at the injured site after intra-arterial transplantation of MSCs [75]. Another study has indicated that MSC transplantation in the ischemic stroke model reduces the size of the damaged area and aid in faster recovery of motor function compared to the control animal model (which did not receive MSC injection) [76].

Moreover, a study using the neonatal mice model of brain damage provided evidence that intraperitoneal intervention of UC-MSCs in a neonatal model significantly reduced the activated microglia and reactive astrocytes in the white matter of neonatal mice brain [77]. Thus, it was suggested that transplanted MSCs play an immunosuppressive role in the neonatal mice model of ischemic brain injury [78]. It is also important to note that UC-MSCs exhibit therapeutic efficacy by modulating the inflammatory processes instead of cell replacement. Collectively, the safety and efficacy of intravenous intervention of UC-MSCs on a small animal model such as neonatal stroke mice, even with a higher dose (1 × 10^5^) of UC-MSCs, were highlighted [47]. Accordingly, Table 1 summarizes some recent studies using animal models of ischemic stroke and mesenchymal stem cell therapy.

Intriguingly, a recent study has reported the use of companion animal model for ischemic stroke injury, since companion animal disease models could better represent the effectiveness and safety of stem cell therapy due to their similarity with human disease, particularly in terms of pathophysiological symptoms, therapeutic responses, gene associations, and biomarkers. Thus, a more effective model was provided for predicting the precise outcomes and risk associated with clinical trials in humans (for instance, the intravenous intervention of MSCs in the cat model for end-stage kidney disorder) [79]. Hence, companion animals could better portray the aftermath of regenerative medicine trials compared to rodent models [80]. Thus, we stated the conceptual advantages of large animal models in translational research, which makes them an attractive model for developing novel endovascular treatments for ischemic brain injury [81]. Nevertheless, large animal model experiments are often more complex than small animal studies. Therefore, training and pilot study plans are necessary for the optimization before the main trial, whereas the time and resource “loss” caused by such preliminary experiments can be atoned by increased reliability and decreased variability in the main trial [81].

Given the therapeutic efficiency of MSCs, we may suggest that MSC treatment could be a promising candidate for bringing a breakthrough in the field of regenerative therapy, especially for central nervous system injury and disease (which severely lack an effective therapeutic strategy) [70]. However, further clinical validation of these studies is required.

## 5. Preclinical and Clinical Trials of MSCs

A growing number of studies from preclinical research suggest stem cell therapy as a promising candidate to treat ischemic brain injury and to reduce its long-lasting effects [82], although the success in phase I clinical trials of stem cell therapy for stroke has significantly enhanced the confidence of the researcher and clinicians for clinical application of stem cell therapy [11]. However, further refinement is required concerning its clinical practicality and to confirm the efficacy and safety of these treatments [83, 84]. Besides, to ensure the solution of ethical, technical, and medical problems before clinical translation, the basic rules underpinning the use of MSCs in clinical trials for stroke patients have been established by the National Institutes of Health Consortium “Stem Cell Therapies as an Emerging Paradigm for Stroke (STEPS)” [68, 85]. In essence, according to STEPS, human trials could either include acute administration of stem cells to minimize the secondary risk of ischemic injury, or it can be a late intervention during the chronic phase of the stroke to augment neuronal regeneration [11].

Moreover, further evidence accumulated from previous clinical trials has suggested the need to improve crucial factors, i.e., appropriate selection of suitable cells and route of administration to effectively translate preclinical results into effective clinical practice [86]. Herein, Table 1 presents a list of recent clinical trials of ischemic brain stroke using mesenchymal stem cell therapy from the ClinicalTrial.gov database (http://www.clinicaltrails.gov). The major limitation of these clinical trials is the small number of patients, which indicates small study effects (Table 2), especially in a single-arm study where the number of samples hardly approaches double figures [36]. However, it is also important to note that early-phase research often includes a smaller size sample; therefore, additional subgrouping seems impractical [83]. Thus, the requirement of a larger sample size for the accurate estimation of stem cell therapy effects was stated[36]. Yet, to date, no study has shown any promising results; therefore, a collaborative effort is a prerequisite to understanding the precise molecular mechanisms representing critical lab-to-clinic translational enabling factors that will lead towards safe and efficient stem cell therapy for the brain ischemic stroke [2].

The development of potency assays during preclinical animal testing is a cardinal aspect before translating cellular therapies into advanced stages of clinical trials. Besides, accurate recognition of safety and efficacy allows it to proceed to phase I/IIa clinical trials [87]. However, it is also important to mention that these clinical trials should first focus on safety confirmation, whereas efficacy endpoints should be next to safety [87].

Moreover, biomarkers with presumed mechanisms of action are also considered critical regulators for late-stage clinical trial approval. Additionally, it has also been recommended to use biomarkers to develop robust, specific, informative, and reproducible potency assays with the potential to describe a fundamental biological effect of the expected benefit [36, 87]. Since the inadequate quality of preclinical tests has been previously considered a major reason for the unsuccessful translation of experimental stroke therapies into the clinic [88, 89], therefore, it is necessary to evaluate and perform preclinical and clinical trials under Good Manufacturing Practice (GMP) before processing it to further clinical trial phases [87].

## 6. Limitations and Overcoming Challenges in Using MSCs for Ischemic Stroke

Notably, in addition to beneficial effects, MSC administration has exhibited some setbacks and side effects for the recipients. Therefore, characterizing the limitations of MSCs activity after their transplantation could aid in identifying a more voracious and comprehensive prospect and role of MSCs in the field of regenerative medicine. Accordingly, here, we highlight some crucial limiting factors of MSCs in the light of previous studies and discuss some aspects to overcome these challenges. Among these factors, the route of administration of stem cells, timing of stem cell delivery, and dose of cells are basic constraints in the clinical translation of stem cell therapy [83]. Although several studies have attempted to investigate the appropriate dose or route of administration of stem cells, yet it is difficult to predict any consequences [36]. Moreover, the correlation of some adverse events such as microocclusion to intra-arterial cell infusion has raised serious safety concerns [66, 88, 90]. Therefore, careful optimization of the intra-arterial infusion procedures should consider before efficacy studies [91, 92], while cell size and infusion velocity also indicate micro-occlusion after intra-arterial cell injection[91]. The fact remains that infusion velocity is also closely related to safe intraarterial administration. Therefore, before planning future preclinical and clinical efficacy studies, careful optimization of cell dose and infusion velocity should be considered on the basis type of stem cells to be delivered [88]. Collectively, it was suggested that appropriate time of MSC administration after the onset of stroke, the optimum dose of cells, and the adequate frequency of stem cell application with precise follow-up could enhance the chances of effective clinical translation of mesenchymal stem cell therapy.

The other concern is the translation of preclinical trials to clinical practice. Despite experimenting with several treatment strategies and various cell types in animal models, their clinical efficacy on stroke patients has not yet been confirmed [93]. The reason for inadequate clinical evidence can attribute to the significant differences in study design between preclinical and clinical trials [62, 94]. The difference in the therapeutic effect of preclinical studies could be due to heterogeneity in infarct size and recipient comorbidities. For instance, <90% of animals used in preclinical trials are reported healthy before stroke induction, whereas many stroke patients suffered from comorbidities such as hypertension, diabetes, and heart disease. Furthermore, stroke patients often take medications such as antidiabetics to counter comorbidities, and these compounds may interact with injected cells [95, 96]. Besides, cell donors' and recipients' ages can influence cell treatment efficacy [97]. Therefore, efficient translation of preclinical study into a successful clinical trial requires the same time window, acute, subacute, or chronic; the same delivery route; the same cell dose (number of cells per kg/body surface area); the same cell immunogenicity; the same preparation procedure before transplantation (e.g., fresh vs. cryopreservation); the same target infarcts (e.g., hemispherical infarcts of middle cerebral artery territory only, with or without reperfusion); matched sex profile; matched age; the same comorbidity; and the same concomitant treatment [93]. Another constrain indicates that age and associated comorbidities such as hypertension and atherosclerosis could alter the vascular constitution and influence the clotting frequency [95]. Therefore, in the future, an optimal protocol of MSC transplantation with enhanced homing and reduced complications is required to be further established and facilitate its translation from bench to clinic [98].

Hence, future comprehensive studies are required to determine the factors, i.e., the optimum time required for isolation, proliferation, characterization of MSCs, and their ability to reach the target site [68, 75]. Besides, it is also crucial to confirm that the intervention of MSCs should not affect the medication or pathophysiology, or illness of patients, and these strategies should pave the way for the application of modified cells [75, 99].

## 7. Future Advancement

The successful outcome of preclinical studies encourages their clinical translation [75, 100]. Accordingly, several bioengineering approaches have assisted in improving the local administration of MSCs. For instance, engineered MSCs are an attractive resource for regenerative therapy due to their specific homing at the target site and their properties to operate with maximum efficiency regardless of the host environment. Besides, a recent study has indicated that identifying host factors that affect the function of MSCs can guide the development of improved MSC application by complementing the host priming strategy for a better therapeutic outcome [101]. However, the clinical application of these recommendations requires further evaluation at the laboratory level to achieve effective clinical trial outcome [101].

Intriguingly, a previous study has shown that multipotent adult progenitor cells administered in patients with acute ischemic stroke exhibited no side effects [102]. However, even after the 90 days of treatment with multipotent adult progenitor cells, no significant improvement in neurological outcomes was observed [102]. Besides, another study demonstrated that intra-arterially delivered autologous bone marrow-derived ALD-401 cells in patients with ischemic stroke did not cause any clinically adverse events in patients with subacute ischemic stroke. However, magnetic resonance imaging (MRI) exhibited a higher incidence of small lesions in the treatment group [103].

Hence, to date, ischemic stroke has no approved treatments to enhance the efficiency of recovery [103]. Besides, a combination of stem cell treatment with interventional procedures has proposed the application of synergistic effects to reinforce the effectiveness of stem cell therapy. Yet, very few good practical conceptions or in-depth studies support this evidence. Further, we will brief some combinational therapies that could be applied to achieve the best clinical outcome.

Among combinational therapies, “Drug-Cell Interaction” holds significant importance [87]. For instance, during clinical practice, patients receiving cell therapy also use medications to deal with stroke comorbidities and secondary prevention. Thus, it was suggested that cell therapy combined with pharmacological treatments enhances its therapeutic effects. However, due to the paracrine effects of stem cells, interactions between drugs and cells cannot be ignored [87]. Therefore, an efficient future perspective for the treatment of ischemic stroke is a prerequisite [87]. Recently, increasing integration of biomaterials has been observed for the efficient delivery of cells to minimize the shear stress caused by needle injections [104, 105] and to improve post-transplantation cell survival [106, 107]. For instance, scaffolds provide structural cues and biochemical signals to support transplanted cells inside the lesion cavity [106, 108]. Systematic optimization of a hydrogel improves the survival and differentiation of human neural stem cells implanted into the stroke-damaged brain [87]. However, scarce evidence indicates the role of combination therapy in functional recovery after ischemic stroke. Moreover, most studies combining biomaterials and cell transplantation are investigatory in nature rather than confirmative studies [87]. Thus suggesting that combinational cell therapies possess the potential to reopen the plasticity time window in chronic stroke, while neurorehabilitation could further aid in recovering the normal functioning of stroke patients [87].

Finally, it is also important to mention that the safety demonstration of multiple preclinical endpoints could be a valuable source upon clinical translation of cellular therapies for stroke treatment. Therefore, a stronger focus on safety rather than confirming efficacy in early preclinical research followed by early safety-oriented clinical research holds the potential to accelerate translational research without compromising the quality [87].

## 8. Conclusion

MSC therapy combined with novel integrative strategies presents an attractive therapeutic modality for treating ischemic stroke. However, due to unsatisfactory clinical outcomes and a lack of understanding about the molecular mechanism, these strategies could not receive approval for their application at the clinical level. Therefore, complete knowledge about molecular events and signaling modalities is required to enhance the efficacy and safety of stem cell therapy. Moreover, developing an efficient therapeutic strategy requires further understanding of the transdifferentiation of transplanted stem cells, their immunomodulatory response, and the mechanism through stem release of growth factors. Nonetheless, cutting-edge technology adapted to high-throughput screening platforms can provide stroke researchers with a mechanism to screen large numbers of potential drug targets in the future. Hence, despite multiple challenges, clinical translation of preclinical studies along with novel therapeutics strategies could make a firm basis for the progression of regenerative medicine in the future.

## Figures and Tables

**Table 1:** Summary of the most recent studies using animal models of ischemic stroke and mesenchymal stem cell therapy.

Sr#	Types of model	Source of stem cells	Time of administration	Dose	Delivery route	Efficacy	Important findings
1	Rats underwent middle cerebral artery occlusion (MCAO) and reperfusion	Mesenchymal stem cells (MSCs)	3-7 days after MCAO	1 × 10^6^ cells/200 UL PBS	Intravenous administration into the tail vein	Enhanced repair to ischemic stroke, through suppression to ischemia-induced microglial activation	This study observed a decreased expression of mincle, a damage-associated molecular pattern (DAMP) receptor, which induces the production of proinflammatory cytokines, suggestive of a potential mechanism in 3D MSC-mediated enhanced repair to ischemic stroke [109]
2	Brain stroke model	Rat (r) MSCs	1 hour after the ischemia/reperfusion	10^6^ ION labeled MSCs in 10 *μ*L saline	Injected into the right CC 1 h after the ischemia/reperfusion procedure	Crosstalk with the CP enhances MSC proliferation and migration in a transwell assay	These findings could shift cell therapy strategies for stroke from intravenous delivery of MSCs to their direct injection into lateral ventricles harboring the CP, which could enhance functional recovery [110]
3	Establishment of transient middle cerebral artery occlusion model	Human cranial bone-derived mesenchymal stem cells (hcMSCs)	3 or 24 h after MCAO	3 or 24 h after MCAO	The cells were administered intravenously through the tail vein	Suppresses the damage of residual nerve cells and leads to functional recovery	This is the first report demonstrating a functional recovery effect after ischemic stroke following hcMSC transplantation [111]
4	Middle cerebral artery occlusion (MCAO)	Conditioned medium (CM) derived from human embryonic MSC (hESC-MSC)	Either one time (1 h post MCAO) or three times (1, 24, and 48 h post MCAO)	5 *μ*L at a flow rate of 0.5 *μ*L/min	Intracerebroventricular	Improved neurogenesis and angiogenesis to accelerate the recovery of cerebral ischemia insult	hESC-MSC-CM remarkably attenuates neurological deficits as well as lesion volume in MCAO rats [112]
5	Mouse model of transient focal cerebral ischemia	Tropomyosin receptor kinase B (TrkB) gene-transfected mesenchymal stem cells (TrkB-MSCs)	Five days after MCAO	1 × 10^6^/2 *μ*L phosphate-buffered saline (PBS)	Injected at an infusion rate of 0.5 *μ*L/min into the peri-infarct site: anteroposterior	TrkB-MSCs promote the expression of BDNF and NT4, induce the differentiation of TrkB-MSCs, and improve motor function	TrkB-MSCs improve motor function in the mouse model [113]
6	Intraluminal middle cerebral artery occlusion (MCAO)	Neural stem cells were isolated from the subventricular zone of the rat brain.	24 hours after local ischemia	5∗10^5^ floating cells in 100 *μ*L of PBS	External carotid artery (ECA) lumen	The transplantation of neural stem cells within 24 hours after ischemia led to a reduction in the neural cells death	Reduction in the neural cell death in the ischemic zone and the brain damage decreased significantly [114]
7	Ischemic stroke mice	Neural stem cells (NSCs)	24 h after MCAO operation	3 × 10^5^ cells	i.v. injection	The BDNF-NSC treatment significantly increased the brain BDNF level	The present study investigates the ROS-responsive charge-reversal polymer B-PDEA as the first successful nonviral vector for effective genetic transfection of NSCs [115]

**Table 2:** Summary of clinical trial study using MSC-based therapy for ischemic stroke.

Sr#	ClinicalTrials.gov identifier	Study type	Type of cell	Type of stroke	Patients demography	Delivery route	Dose safety & efficacy	Study objectives
1	NCT01019733	Phase n/a interventional	Autologous hematopoietic (CD34+) cells	Hypoxic/ischemic brain injury	18 participants of 1 year to 8 years (child) of all sexes	Intrathecal administration	5 to 10 mL of stem cells will be infused intrathecally	An overall 4.7-month increase in developmental age according to the Battelle Developmental Inventory PM
2	NCT04590118	Phase randomized open-label	Allogeneic umbilical-cord mesenchymal stem cells	Ischemic stroke	18 years or older clinical diagnosis of ischemic stroke for more than 6month	Single intravenous infusion of it-hMSC for ischemic stroke patients	Single intravenous infusion of 0.5 × 10^6^, 1 × 10^6^, and 2 × 10^6^ hMSC/kg	The purpose of this study is to evaluate the safety, tolerability, and preliminary efficacy of a single injection of it-hMSC in patients with ischemic stroke in a multicenter, blind, randomized, placebo-controlled trial
3	NCT03186456	Phase I/II interventional	Allogeneic umbilical-cord mesenchymal stem cells	Cerebral infarction	18 years or older, diagnosis of more than 6 months exhibiting functional deficit	Intravenous infusion	Single intravenous infusion of 0.5 × 10^6^, 1 × 10^6^, and 2 × 10^6^ it-hMSC/kg	A phase I/IIa study to evaluate the safety, tolerability, and preliminary efficacy of a multicenter, blind, randomized, placebo-controlled single injection of it-hMSC in patients with ischemic stroke
4	NCT03176498	Phase I/phase I, nonrandomized	Allogeneic umbilical-cord mesenchymal stem cells	Cerebral infarction	20 years to 75 years, proved cerebral infarction by CT or MRI.	Intravenous infusion	The experimental group received allogeneic umbilical cord mesenchymal stem cell and aspirin enteric-coated tablets, 0.1 g/d by mouth	The effects of human umbilical cord mesenchymal stem cell therapy on neurological function for cerebral infarction patients in convalescent period
5	NCT04097652	Phase I randomized	Human umbilical cord-derived mesenchymal stem cells (UMC119-06)	Transient ischemic attack	9 patients with 20 years to 80 years onset of ischemic stroke within 48 to 168 hours before the start of treatment	Intravenous infusion.	Low does to a high dose of UMC119-06 with three months of time frame	The safety and tolerability after intravenous infusion of UMC119-06 in subjects with acute ischemic stroke
6	NCT04093336	Phase I/II nonrandomized	Human umbilical cord mesenchymal stem cells	Cerebral infarction/acute ischemic stroke	Patients aged 18~80 years with a clinical diagnosis of ischemic stroke for more than 6 months	Intravenous transplantation	Intravenous MSCs 2 × 10^6^/kg as a single dose and standardized treatment of acute ischemic stroke	Effect of human umbilical cord mesenchymal stem cell (MSC) transplantation for prognosis of acute cerebral infarction patients
7	NCT01310114	Phase II randomized	Human placenta-derived cells (PDA001)	Acute ischemic stroke	Patients of all sexes with 18 years to 80 years of age	Intravenous infusion	2 × 10^8^ cells in 240 mL per infusion	A phase 2A, prospective, multi-center, randomized, double-blind, placebo-controlled, dose-escalation study to evaluate the safety of intravenous infusion of human placenta-derived cells (PDA001) for the treatment of adults following ischemic stroke
8	NCT01297413	Phase I/II, randomized open-label	Allogeneic adult mesenchymal bone-marrow stem cells	Ischemic stroke	18 years and older with clinical diagnosis of ischemic stroke for longer than 6 months	Intravenous infusion	Intravenous one dose of 0.5-1.5 million cells per kg of allogeneic adult mesenchymal bone marrow stem cells	A phase I/II, multicenter, open-label study to assess the safety, tolerability, and preliminary efficacy of a single intravenous dose of allogeneic mesenchymal bone marrow cells to subjects with ischemic stroke
9	NCT02580019	Phase II randomized	Human umbilical cord mesenchymal stem cells	Ischemic stroke	18 years to 70 years confirmed diagnosis of intracerebral ischemic stroke in three months by magnetic resonance imaging(MRI)	Intravenous infusion	A single dose of 2 × 10^7^ hUC-MSC will be treated to patients	Cell therapy by intravenous injection of umbilical cord derived mesenchymal stem cells after stroke

## Abbreviations

GFAP: Glial Fibrillary Acidic Protein
JNK: Pathway c-Jun N-terminal kinase pathway
p38: Protein 38
MAPK: Mitogen-Activated Protein Kinase
PI3K: Phosphatidylinositol-3-Kinase
Akt: Akt kinase or protein kinase B or PKB
ERK1/2: Extracellular signal-regulated kinases 1 and 2
rtPA: Recombinant tissue plasminogen activator
Wnt: Wnt/*β*-catenin signaling pathway
NeuN: Neuronal nuclear protein
MAP-2: Microtubule-associated protein-2
BMSCs: Bone marrow stem cells
UC-MSCs: Umbilical cord-derived mesenchymal stem cells
BBB: Blood-brain barrier
CAMs: Cell adhesion molecules.
